# Validation of the Schizophrenia Quality of Life Scale Revision 4 (SQLS-R4) Among Patients with Schizophrenia

**DOI:** 10.3889/oamjms.2016.015

**Published:** 2016-01-11

**Authors:** Viktor Isjanovski, Andromahi Naumovska, Dimitar Bonevski, Antoni Novotni

**Affiliations:** 1 *Psychiatric Hospital “Skopje”, Skopje, Republic of Macedonia*; 2 *Psychiatric Clinic, Faculty of Medicine, Ss Cyril and Methodius University of Skopje, Skopje, Republic of Macedonia*

**Keywords:** schizophrenia, patients, reliabity, validity, the Quality of Life Scale

## Abstract

**BACKGROUND:**

The main goal of psychiatric care is not to be focused only on reducing psychopathological symptoms, but on improvement of the patient’s quality of life.

**AIM:**

To examine validation and reliability SQLS-R4 among patients with schizophrenia.

**METHODS:**

The sample consisted of 61 outpatients with schizophrenia attending the Psychiatry Hospital “Skopje”. nclusion criteria for subject selection were: 1) age more than 18 years, 2) clinically stable (not acutely ill or has not been recently hospitalized at least for the past 3 months). They completed SQLS-R4 and SF36 questioners.

**RESULTS:**

The internal consistency reliability was satisfactory for both the psychosocial and vitality domains (Cronbach’s α = 0.928, 0.83). Most of the items were significantly correlated with own scale score (from 0.189 to 0.687). The average of the score for the psychosocial quality life was 39.9 ± 8.6 (sometimes), for the cognition and vitality was 26.5 ± 6.1 (sometimes) (SQLS-R4). There was moderate correlation between SF 36-energy with SQOLS - motivation and energy; SF 36-mental health correlation with SQOLS-psychosocial.

**CONCLUSION:**

SQLS-R4 appears to offer excellent potential as an easily administered and patient acceptable assessment and monitoring measure of quality of life (QoL). However, a principle psychometric criterion crucial to the use and validity of the instrument concerns the underlying factor structure.

## Introduction

Schizophrenia is a serious mental disorder characterized by loss of contact with reality-psychosis, hallucinations, delusions, abnormal thinking, irrational thinking, bizarre behaviour, restrictive range of emotions, motivation weakened and impaired social and work function, chronic mental illness that differently affect the behaviour, thought and emotion [1-3]. Nowadays, the main goal of psychiatric care is not to be focused only on reducing (the) psychopathological symptoms, but (one part of the main goal of psychiatric care is the) improvement of the patient’s quality of life. Decreased quality of life is often an important cause or consequence of psychiatric illness, and needs to be included in a comprehensive treatment plan. Literature research reveals that mentally ill patients declare (a) lower overall quality of life compared to the general population [4-9]. People with schizophrenia are at increased risk for multiple and chronic social, cognitive, and behavioural deficits that may lead to inadequate health and self-care practices [7-10]. Quality of live may be defined as a person’s sense of wellbeing and satisfaction with his/her life circumstances, as well as a person’s health status and access to resources and opportunities [11-12].

Many instruments have been developed to measure HRQOL (health-related quality of life), but there is no consensus on the most appropriate scale for measuring HRQOL in schizophrenia [13]. The instrument of choice for HRQOL measurement depends on the assessment purposes [14]. Generic instruments designed to be applicable across all diseases or conditions are likely to be useful in comparing different group of patients, while disease-specific measures have more potential in detecting treatment effects [15].

The hypotheses are: H1 - The SF 36 energy dimension would be strongly associated with SQOLS motivation and energy dimension; and H2 - The SF 36 mental health dimension would be strongly associated with SQOLS psychosocial score.

The aim of this study is validation of the Schizophrenia Quality of Life Scale Revision 4 (SQLS-R4) among patients with schizophrenia. This study was designed to determine the reliability and validity of the Quality of Life Scale Revision 4 (SQLS-R4) for schizophrenic patients, and to compare SQLS-R4 with short-term change in SF-36 scores for patients.

## Material and Methods

### Subjects

Of the 70 people with schizophrenia who were approached, 61 (87.1%) agree to take part, sample of 61 outpatients with schizophrenia attending the Psychiatry Hospital “Skopje”. Inclusion criteria for subject selection were: 1) age more than 18 years, 2) clinically stable (not acutely ill or has not been recently hospitalized at least for the past 3 months). The mean age of the patients was 47.4 years (s.d. 9.0, min.-32, max-74) (Table 1 & Fig.1).

**Table 1:** Corrected item to total correlations (p) and internal reliability (Cronbach`s α) of scales

Scale and items	Corrected Item-Total Correlation	Cronbach’s α
Psychosocial P 3	.527	0.928
P 4	.548	
P 5	.580	.
P 6	.563	
P 8	.535	
P 10	.630	
P 11	.541	
P 13	.687	
P 15	.529	
P 16	.640	
P 17	.528	
P 18	.568	
P 19	.506	
P 21	.602	
P 22	.611	
P 24	.607	
P 25	.554	
P 27	.517	
P 29	.662	
P 30	.634	
Cognition and vitality		0.83
P 1	.686	
P 2	.675	
P 7	.330	
P 9	.522	
P 12	.456	
P 14	.180	
P 23	.381	
P 20	.560	
P 26	.400	
P 28	.377	
P 31	.572	
P 32	.626	
P 33	.565	

### Measure

The internal consistency of the SQLS-R4 was determined by item to total correlations and Cronbach’s alpha coefficients [16, 17].

The SQLS-R4 was developed by Wilkinson et al. [18-20] for the measurement of HRQOL in people with schizophrenia. It comprises 33 items incorporated in two domains: psychosocial feelings (22 items) and cognition and vitality (11 items). All except four items are scored on a five-point Likert-type scale (0- never, 1-rarely, 2- sometimes, 3- often, 4- always), with the exceptional four items being reverse coded (0- always, 1-often, 2- sometimes, 3- rarely, 4- never). Individual domain and total scores are standardized by scoring algorithm to a 0 (best health status) to 100 (worst health status) scale, with higher scores indicating comparatively lower quality of life. All questionnaires were scored using a Likert-type format. The SQLS-R4 was completed by all patients within 5-10 min, the few who took longer expressed the need to think longer about their responses. The instrument was translated from English to Macedonian using the procedure of forward backward translation procedure. With intention to develop a Macedonian version of the instrument that is conceptually equivalent to the original version, the back-translated English version was compared and discussed.

The SF-36 consists of 36 items divided into 2 components: the Physical Component and the Mental Component. The final scores of each dimension ranges from 0-100, with the highest score corresponding to a better condition. The physical component includes the following dimensions: Physical Functioning (PF), Physical Role Functioning (RP), Bodily Pain (BP) and General Health (GH). The Mental Component is measured by the following dimensions: Vitality (VT), Mental Health (MH), Social Functioning (SF) and Emotional Role Functioning (RE). These eight dimensions also can be used to generate a physical and mental health summary score [21].

### Statistical analysis

Patient characteristics were presented by using descriptive statistics. The construct validity was established by comparing scores of the SQLS-R4 subscales with those of the SF 36, by Spearman`s rank correlations. The level of statistical significance was defined less than 0.05. The statistical analyses were performed by using SPSS version 20.0

## Results

Completed questionnaires were collected from 61 patients suffering from schizophrenia.

Table 1 shows the correlations of items with their scale totals, and the internal consistency reliability of the scales (that is the extent to which items in a scale reflect a single underlying dimension). Items were highly correlated with their own scale score (corrected to exclude the item being correlated). Internal reliability was assessed using Cronbach α statistic. The internal consistency reliability was satisfactory for both the psychosocial and vitality domains (Cronbach’s α = 0.928, 0.83), the deletion of any of the 33 items did not improve the Cronbach α value for booth domains.

For some items correlations were satisfactory for the psychosocial domains ranging from 0.506-0.687 and the vitality domains -0.456 - 0.686. Some items from the vitality subscale had correlations less than 0.39 (p23 = 0.381; p28 = 0.377; p7 = 0.333; p14 = 0.189). Most of the items were significantly correlated with own scale score (from 0.189 to 0.687) (Table 1).

Table 2 shows the average of the score for the psychosocial quality life was 39.9 ± 8.6 (sometimes), for the cognition and vitality was 26.5 ± 6.1 (sometimes) each scale has a range from 0-best possible health to 100-worst possible health.

**Table 2:** Descriptive Statistics

	N	Minim.	Maxim.	Mean	Std. Deviation	Skewness		Kurtosis	
						Statistic	Std. Error	Statistic	Std. Error
Quality life	61	28.00	94.00	66.4754	13.69502	-.467	.306	.612	.604
Psychosocial quality life	61	17.00	57.00	39.9508	8.57793	-.425	.306	.550	.604
Cognition vitality quality life	61	10.00	38.00	26.5246	6.05422	-.524	.306	.258	.604

Table 3 presents the demographic profile of the patients. The mean age of the patients sample was 47.4 years. A majority of patients were secondary school educated - 70.5%. In the study participated- 68.9% male and 31.15 female, 36.1%of the patients were living alone and majority of them - 68.9% were single, most of them were unemployed - 65.6%.

**Table 3:** Demographic profile of survey population

**Gender**	%	***N***

Male	68.9	42
Female	31.1	19

**Age**	**mean ± SD**	
	47.4 ± 8.9 y	
**Education**	%	***N***

Primary school	16.4	10
Secondary school	70.5	43
University	13.1	8

**Marital status**	%	***N***

Single	68.9	42
Married	31.1	18

**Living arrangement**	%	***N***

With family or relatives	63.9	39
Living alone	36.1	22

**Employment status**	%	***N***

**Employed**	34.3	21
**Unemployed**	65.6	40

**Duration of illness**	**mean ± SD**	
	18.1 ± 9.4 y	

Construct validity was assessed comparing results on the SF36 - The Short Form [36] Health Survey. The predict correlations were substantiate- SF 36-energy correlation with SQOLS- motivation and energy ρ=0.63 for p<0.05; SF 36-mental health correlation with SQOLS-psychosocial ρ=0.52 for p<0.05, it was moderate correlation (Correlation coefficients-Spearman) (Table 4).

**Table 4:** Correlations between the SQOLS and SF 36

	SQOLS-motivation and energy	SQOLS-psychosocial
SF 36-energy	ρ=0.63	
SF 36-mental		ρ=0.52

## Discussion

The internal consistency reliability was satisfactory for both the psychosocial and vitality domains in our study were high (Cronbach’s α = 0.928, 0.83). Cronbach’s α was also high as those found in Taiwan [22], Malaysia [23] and UK study [18]. The internal consistency reliability for the psychosocial and vitality domains in Taiwan study [7] were very similar - Cronbach’s α = 0.92 and 0.84; and in Malaysia study [23] - Cronbach’s α = 0.95 and 0.85.

The characteristics of the SQOLS-R4 scale were good. Assessment of the internal consistency revealed significantly high correlations of items with their scale total except for four items.

Similar findings were also found in the validation of the SQOLS-R4 in the Japan study by Kaneda Y. et al [24] and Malaysia study by Nur Akmar Taha et al [23]. In Malaysia study [23] validity was supported by correlations between domains measuring related constructs of the SQO LS-R4 and SF-36 (ρ - 0.65 to 0.67), the correlations which was obtained in our study confirms (ρ - 0.63 to 0.52) findings. As suggested by Kuo et al. [22], these results may be indicative of those items being interpreted differently from the vitality construct in the Asian customs. Alternatively, because of the study design, kind of patients (patients with various degree of symptom severity), kind of symptoms (negative and positive, depressive, cognitive) and sample size. The sample size for both studies in the Taiwan and Japan was relatively smaller than study in Malaysia and our sample size was smallest than other tree study. It was hypothesized that significant correlations between scores would be found for the SF-36 with all dimensions of the SQOLS. These hypothesized associations were indeed found.

Construct validity was explored by correlation of the scales of the SQLS with established psychiatric self-report measures and the SF-36. Results suggest that the measure is addressing areas related, but not identical, to those of previously existing measures. The SQLS was developed to be a valid and feasible questionnaire for self-completion that addresses the perceptions and concerns of people with schizophrenia - except, of course, those too unwell to complete the questionnaire. Its main use is likely to be in clinical trials and the evaluation of clinical interventions. Evidence is presented in this report to suggest that the SQLS has desirable properties in terms of reliability and validity, and we have found the measure to have excellent acceptability and feasibility in practice.

The background characteristics of the study patients were: most of them were male; a majority of them were aged approximately 47 years with mean illness duration of 18 years; marital status-single, unemployed, and education status-secondary school.

In conclusion, quality of life is a major area of concern for patients with chronic schizophrenia. The schizophrenia quality of life scale revision 4 (SQOLS-R4) appears to offer excellent potential as an easily administered and patient acceptable assessment and monitoring measure of quality of life. The questionnaire was found to be acceptable total patients and feasible for use in a routine clinical setting.

## Appendix

Items (p) in the SQOLS

1. Lack energy to do things
2. Can t be bothered to do things?
3. Worry about future
4. Feel lonely
5. Feel hopeless
6. Feel panicky
7. able to carry out daily activities
8. Take things people say the wrong way
9. Find hard to concentrate
10. difficult to mix with people
11. Feel down
12. Feel I can cope
13. Feel very mixed up
14. Sleep well
15. Feelings go up and down
16. Concerned won’t get better
17. Worry about things
18. Feel people avoid me
19. Get upset thinkking about the past
20. Trouble remembering things
21. Feel cut off from the world
22. Feel uncomfortable with people
23. Has trouble thinking clearly
24. Has upsetting thoughts
25. Have suicidal thoughts
26. Feel happy
27. Feel depressed
28. Feel drowsy
29. Feel restlessness
30. Concerned about the social life
31. Feel tired
32. Feel physical weak
33. Feel like not leading a normal life
